# Validation of bifurcation DEFINITION criteria and comparison of stenting strategies in true left main bifurcation lesions

**DOI:** 10.1038/s41598-020-67369-9

**Published:** 2020-06-26

**Authors:** Juan Wang, Changdong Guan, Jue Chen, Kefei Dou, Yida Tang, Weixian Yang, Yanpu Shi, Fenghuan Hu, Lei Song, Jiansong Yuan, Jingang Cui, Min Zhang, Shuang Hou, Yongjian Wu, Yuejin Yang, Shubin Qiao, Bo Xu

**Affiliations:** 1 Department of Cardiology, Fu Wai Hospital, National Center for Cardiovascular Diseases, Chinese Academy of Medical Sciences, Beijing, China; 2 Catheterization Laboratories, Fu Wai Hospital, National Center for Cardiovascular Diseases, Chinese Academy of Medical Sciences, Beijing, China; 3 National Clinical Research Center for Cardiovascular Diseases, A 167, Beilishi Road, Xicheng District, Beijing, 100037 China; 4 CCRF (Beijing) Inc, Beijing, China

**Keywords:** Interventional cardiology, Cardiovascular diseases, Coronary artery disease and stable angina, Ischaemia

## Abstract

There are controversies on optimal stenting strategy regarding true left main (LM) bifurcation lesions. The present study compared 1- and 2-stenting strategy for patients with true LM bifurcation lesions as differentiated by DEFINITION criteria. 928 patients with true LM bifurcation lesions (Medina 1,1,1 or 0,1,1) treated with DES were enrolled consecutively. 297 (32.0%) patients were identified as complex LM bifurcation, and 631 (68.0%) patients into simple LM bifurcation group according to DEFINTION criteria. Patients in complex vs. simple LM bifurcation group had significantly higher major adverse cardiac event (MACE, including cardiac death, myocardial infarction [MI] and ischemia-driven target vessel revascularization) rate at 30 days (7.8% vs. 4.0%, p = 0.01), 1 year (10.3% vs. 6.4%, p = 0.04), and numerically at 3 years (14.2% vs. 10.1%, p = 0.07), which was mainly driven by increased MI. Moreover, patients in the 2-stent strategy group had strong trend towards lower incidence of cardiac death in both complex LM bifurcation group (2.0% vs. 5.9%, p = 0.08) and simple LM bifurcation group (1.9% vs. 4.5%, p = 0.07). In conclusion, the complex bifurcation lesion criteria established in DEFINITION study was able to risk-stratify LM bifurcation patients. Two-stent technique yielded numerically lower 3-year cardiac mortality regardless of LM bifurcation complexity.

## Introduction

Percutaneous coronary intervention (PCI) for bifurcation lesions, particularly those in the left main (LM) coronary artery, carries the risk of potential acute occlusion of side branches (SB) and higher rates of in-stent restenosis events^1–3^. It comes to an agreement that for treatment of non-LM bifurcation lesions, the simpler provisional stenting strategy is safe and clinically impactful^4–8^. However, there are controversies on optimal stenting strategy regarding LM bifurcation lesions, especially after good clinical benefits revealed by double-kissing (DK) crush 2-stent strategy^9^. There are limitations in previous studies that patients were not stratified based on lesion complexity. Since 2-stent strategy normally performed in more complex LM bifurcations with specific anatomy, which will lead to a better result in favors of single-stent strategy. The present study sought to investigate whether DEFINITION criteria^10^ (true bifurcation lesions in LM, or with large SB, severe SB plaque burden, moderate to severe calcification and multiple lesions, and longer or diffuse main vessel lesions indicate complex bifurcation lesions) could identify lesion complexity for true LM bifurcation lesions (Medina type 1,1,1 or 0,1,1) and compare 1- and 2-stenting strategy for the treatment of true LM bifurcation lesions as differentiated by DEFINITION criteria in a large serial cohort of LM-PCI population.

## Methods

### Population

Between January 2004 and December 2015, 928 patients with true LM bifurcation lesions (Medina type 1,1,1, or 0,1,1) treated with PCI at a large center (Fu Wai Hospital, Chinese Academy of Medical Sciences, Beijing, China) were consecutively enrolled. Patients with acute myocardial infarction (MI) within 72 h were excluded. Clinical and procedural characteristics were prospectively recorded in a dedicated database. Features of main vessel (MV)/SB lesion as listed in DEFINITON criteria were retrospectively evaluated by an independent core laboratory (Interventional Cardiovascular Imaging Core Laboratory, National Center for Cardiovascular Diseases, Beijing, China). Bifurcation lesions were classified according to the Medina classification^11^, in which MV and SB components of the bifurcation are assigned depending on the presence or absence of a stenosis > 50%. Bifurcation angle was defined as the angle between MV and SB measured from mid vessel to mid vessel^12^. Clinical follow-up visits at 1 month, 1 year, and annually thereafter up to 3 years were at an independent office and all adverse clinical events were evaluated and adjudicated by an independent physician group who were not involved in the index PCI procedures.

The present study was approved by the institutional review board at Fu Wai hospital. The study was conducted in accordance with the Declaration of Helsinki. All eligible patients provided informed consent for long-term follow up by telephone or clinic visit after the index procedure.

### Procedures

Coronary angioplasty and PCI procedures were performed according to standard techniques, stent type and use of intravascular imaging (intravascular ultrasound or optical coherence tomography) was at physician’s discretion. Individual doctors decided treatment strategy, e.g., 1- or 2-stent technique. A provisional stenting approach was recommended for LM treatment in most cases, while the elective treatment of LM bifurcation lesions with involvement of both the MV and the SB is more likely to require a 2-stent approach. As a retrospective study, patients who received a bail-out stenting due to unplanned deterioration following provisional stenting strategies were categorized into 2-stent group. Final kissing balloon dilation was recommended for 2-stent but not 1-stent strategy, and choice among 2-stent techniques (T-stenting, V-stenting, crush and its modifications, culotte, among others) was left to the discretion of the operators based on their clinical experience. All patients received 100 mg aspirin and 75 mg clopidogrel once daily for at least 6 days; otherwise, a loading dose of aspirin (300 mg) and clopidogrel (300 mg) was required. After PCI procedure, patients were maintained on aspirin (100 mg once daily) indefinitely and clopidogrel (75 mg once daily) for at least 1 year following drug-eluting stent (DES) implantations; any changes to adjunctive pharmacotherapy were at operator’s discretion.

### DEFINITION criteria

According to DEFINITION criteria^10^, complex LM bifurcation lesions were defined as those meeting a major risk factor: SB diameter stenosis ≥ 70% and SB lesion length ≥ 10 mm, or any 2 minor risk factors: moderate to severe calcification, multiple lesions, bifurcation angle < 45°, main vessel reference vessel diameter < 2.5 mm, thrombus-containing lesions, and MV lesion length ≥ 25 mm.

### Outcomes and definitions

Cardiac death was defined as any death due to cardiac cause (e.g. MI, low-output failure, fatal arrhythmia), unwitnessed death and death of unknown cause, and all procedure related deaths including those related to concomitant treatment. Periprocedural MI was defined as creatine kinase concentration > 2 times the upper limit of normal and stent thrombosis as any definite or probable Academic Research Consortium defined thrombosis^13^. Target vessel revascularization (TVR) was defined as any revascularization within the entire major coronary vessel including downstream of the main vessel as well as side branches. A composited endpoint of major adverse cardiac events (MACE) as defined in DEFINITON study was also investigated, which including cardiac death, MI, or TVR.

### Statistical analysis

Categorical variables are reported as percentage (counts) and were compared using chi-square or Fisher exact test. Continuous data are presented as mean ± SD and were compared using a 2-sample *t* test. A p value < 0.05 was considered as statistically significant. In order to reduce the possible selection bias between 1- or 2-stent groups an inverse-probability-of-treatment weighting (IPTW) method is used. The propensity scores are estimated by multiple logistic regression analysis that included all patient demographic as well as lesion and procedural characteristics listed in SI Table S1. Model discrimination was assessed with *c*-statistics, and baseline characteristics of patients after IPTW adjustment are presented as standardized difference. Three-year outcomes before or after IPTW adjustment are presented as Kaplan–Meier estimates and compared using log-rank test as well as Cox regression model. All statistical analyses were performed with SAS 9.4 (Cary, NC, United States).

## Results

### Baseline characteristics

**Figure 1:**
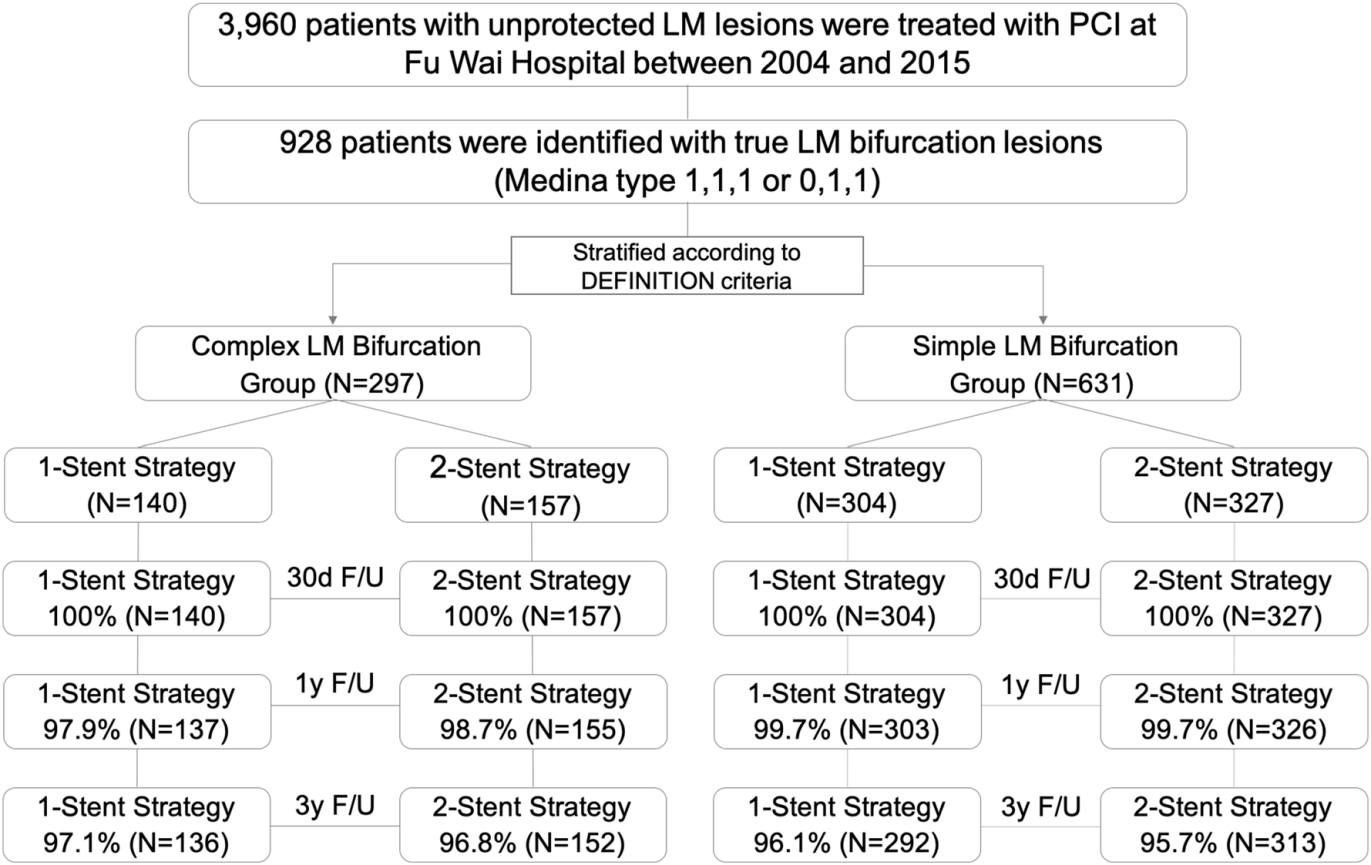
Patient FLOW. Thirty-day follow-up includes a window of ± 7 days, 1- and 3-year follow-up includes a window of ± 30 days. *LM* left main, *PCI* percutaneous coronary intervention, *F/U* follow-up.

Among the 928 true LM bifurcation patients, 297 (32.0%) patients were stratified into complex LM bifurcation group according to DEFINITION criteria, and 631(68.0%) patients into the simple LM bifurcation group. In complex bifurcation group, 140 vs. 157 patients were treated with 1- or 2-stent strategy, respectively. On the other hand, 304 vs. 327 patients were treated with 1- or 2-stent strategy in the simple LM bifurcation group (Fig. 1).

Patients in the complex vs. simple LM bifurcation group were older (62.0 vs. 60.3 years, p = 0.02) and more likely presented with a comorbid condition as well as complex lesion anatomy (SI Tables S2, S3). While baseline information for patients treated with 1- or 2-stent strategy in simple or complex LM bifurcation group were similar (Table 1), expect more Medina type 1,1,1 lesions received 2-stent strategy (92.4% vs.84.3%, p = 0.03 in complex LM bifurcation group; 84.7% vs.66.8%, p < 0.001 in simple LM bifurcation group), and lesion seems longer in complex LM bifurcation group receiving 2-stent strategy but inversely in simple LM bifurcation group, also large side branches with higher diameter stenosis tends to be treated with 2-stent strategy (Table 2). Since 2-stent strategy requiring more steps and sometimes challenging to perform, there were more post-dilation, intra-aortic balloon pump utilization as well as more intravascular ultrasound guidance as shown in Table 3.

**Table 1:** Baseline patient characteristics between 1- or 2-stent strategy by LM bifurcation group.

	Complex LM bifurcation group			Simple LM bifurcation group		
	1-stent strategy (N = 140)	2-stent strategy (N = 157)	p	1-stent strategy (N = 304)	2-Stent strategy (N = 327)	p
Age, years	62.2 ± 9.9	61.8 ± 9.9	0.83	60.3 ± 10.8	60.4 ± 11.0	0.85
Male	77.1% (108)	82.8% (130)	0.22	81.9% (249)	78.6% (257)	0.30
Body mass index, kg/m^2^	25.5 ± 3.1	26.0 ± 3.1	0.14	25.9 ± 3.2	25.6 ± 3.2	0.23
Diabetes mellitus	27.1% (38)	31.2% (49)	0.44	26.0% (79)	30.6% (100)	0.20
Insulin-requiring	2.9% (4)	3.8% (6)	0.65	3.6% (11)	3.4% (11)	0.86
Hypertension	56.4% (79)	62.4% (98)	0.29	56.6% (172)	57.2% (187)	0.88
Hyperlipidemia	57.9% (81)	59.9% (94)	0.72	54.0% (164)	51.7% (169)	0.57
Family history of coronary artery disease	20.7% (29)	16.6% (26)	0.36	18.4% (56)	15.9% (52)	0.40
Current tobacco use	29.3% (41)	34.4% (54)	0.35	35.2% (107)	33.9% (111)	0.74
Previous myocardial infarction	37.1% (52)	30.6% (48)	0.23	31.6% (96)	28.8% (94)	0.44
Previous stroke	10.7% (15)	10.2% (16)	0.88	9.5% (29)	6.7% (22)	0.20
Peripheral arterial disease	7.1% (10)	7.6% (12)	0.87	3.0% (9)	4.0% (13)	0.49
Previous percutaneous coronary intervention	32.1% (45)	29.9% (47)	0.68	27.0% (82)	30.0% (98)	0.41
Creatinine clearance rate	86.6 ± 27.2	92.1 ± 26.5	0.09	90.9 ± 28.5	90.6 ± 25.1	0.62
Unstable angina	59.3% (83)	58.0% (91)	0.82	62.8% (191)	68.8% (225)	0.11
Left ventricular ejection fraction, %	61.6 ± 8.9	61.4 ± 8.1	0.87	62.5 ± 7.8	63.2 ± 7.1	0.38
**Coronary artery disease extent**			0.43			0.10
Isolated LM	0% (0)	0% (0)		1.6% (5)	0.3% (1)	
LM + 1VD	1.4% (2)	0% (0)		4.6% (14)	2.1% (7)	
LM + 2VD	37.9% (53)	37.6% (59)		47.0% (143)	51.4% (168)	
LM + 3VD	60.7% (85)	62.4% (98)		46.7% (142)	46.2% (151)	
SYNTAX Score	29.1 ± 6.7	27.7 ± 5.4	0.10	25.3 ± 7.8	26.1 ± 6.0	0.30

Values are mean ± SD or % (n). *Multiple lesions included multiple-vessel disease (defined as ≥ 70% stenosis in at least 1 major epicardial vessel and ≥ 50% stenosis in at least 1 other major vessel) or ≥ 2 lesions separated by at least a 5-mm normal segment in the target vessel.

*LM* left main, *VD* vessel disease, *SYNTAX* synergy between percutaneous coronary intervention with TAXUS and cardiac surgery.

**Table 2:** Baseline lesion characteristics between 1- or 2-stent strategy by LM bifurcation group.

	Complex LM bifurcation group			Simple LM bifurcation group		
	1-stent strategy (N = 140)	2-stent strategy (N = 157)	P	1-stent strategy (N = 304)	2-stent strategy (N = 327)	p
**Classification**			0.03			< 0.0001
Medina 1,1,1	84.3% (118)	92.4% (145)		66.8% (203)	84.7% (277)	
Medina 0,1,1	15.7% (22)	7.64% (12)		33.2% (101)	15.3% (50)	
Chronic total occlusion	6.4% (9)	3.8% (6)	0.31	3.9% (12)	1.2% (4)	0.03
Moderate to severe calcification	25.7% (36)	25.5% (40)	0.96	8.6% (26)	5.2% (17)	0.09
Thrombus-containing	6.4% (9)	2.6% (4)	0.10	1.0% (3)	0.3% (1)	0.62
Multiple lesions*	100.0% (140)	100.0% (157)	NA	100.0% (304)	99.1% (324)	0.25
**Main vessel**						
Lesion length, mm	37.2 ± 16.4	41.5 ± 22.1	0.29	22.4 ± 15.0	20.0 ± 14.8	0.09
Lesion length ≥ 25 mm	80.7% (113)	87.9% (138)	0.09	29.9% (91)	14.4% (47)	< 0.0001
Reference vessel diameter, mm	3.43 ± 0.45	3.53 ± 0.54	0.29	3.61 ± 0.46	3.76 ± 0.53	0.0006
Reference vessel diameter < 3.0 mm	7.9% (11)	5.7% (9)	0.47	4.3% (13)	2.2% (7)	0.13
Reference vessel diameter < 2.5 mm	1.4% (2)	0% (0)	0.22	0% (0)	0% (0)	NA
Diameter stenosis, %	87.9 ± 8.1	84.9 ± 8.5	0.003	84.4 ± 10.4	81.8 ± 10.7	0.002
Diameter stenosis ≥ 70%	99.3% (139)	97.5% (153)	0.37	94.7% (288)	92.7% (303)	0.28
**Side branch**						
Lesion length	28.1 ± 17.4	33.3 ± 24.9	0.24	21.9 ± 15.2	18.9 ± 16.7	0.0002
Lesion length ≥ 10 mm	100.0% (140)	100.0% (157)	NA	86.2% (262)	79.5% (259)	0.03
Reference vessel diameter	2.80 ± 0.37	3.01 ± 0.43	0.0002	2.94 ± 0.41	3.07 ± 0.42	0.0002
Reference vessel diameter ≥ 2.5 mm	93.6% (131)	100.0% (157)	0.001	97.4% (296)	97.6% (318)	0.89
Diameter stenosis, %	85.7 ± 9.9	84.6 ± 8.1	0.38	69.4 ± 16.9	78.5 ± 12.5	< 0.0001
Diameter stenosis ≥ 70%	100.0% (140)	100.0% (157)	NA	55.6% (169)	85.0% (278)	< 0.0001
Bifurcation angle < 45°	2.7% (3)	2.4% (3)	1.00	1.4% (3)	0.5% (1)	0.37

Values are mean ± SD or % (n). Abbreviations as in Table 1.

**Table 3:** Procedural characteristics and results.

	Complex LM bifurcation group			Simple LM bifurcation group		
	1-stent strategy (N = 140)	2-stent strategy (N = 157)	p	1-stent strategy (N = 304)	2-stent strategy (N = 327)	p
Transradial approach	60.7% (85)	63.7% (100)	0.60	69.4% (211)	54.1% (177)	< 0.0001
Guidance with IVUS	30.7% (43)	51.6% (81)	0.0003	27.6% (84)	55.4% (181)	< 0.0001
**Stent implantation**						
Number of stents per patient	2.45 ± 1.15	3.25 ± 1.06	< 0.0001	1.75 ± 0.95	2.56 ± 0.97	< 0.0001
Stent diameter, mm	3.20 ± 0.42	3.32 ± 0.47	0.05	3.44 ± 0.50	3.41 ± 0.48	0.37
Stent length, mm	43.8 ± 19.5	51.3 ± 26.6	0.03	29.0 ± 16.9	30.1 ± 18.9	0.27
Maximum inflation pressure, atm	15.6 ± 3.0	16.3 ± 2.9	0.001	15.6 ± 2.9	16.1 ± 3.1	0.08
2-stent strategy	0% (0)	100.0% (157)	NA	0% (0)	100.0% (327)	NA
Crush	–	76.4% (120)		–	66.7% (218)	
Mini crush		59.9% (94)			54.7% (179)	
DK crush		16.6% (26)			11.9% (39)	
T-stent	–	9.6% (15)		–	12.8% (42)	
V- or kissing stent	–	4.5% (7)		–	11.9% (39)	
Culotte	–	9.6% (15)		–	8.6% (28)	
Final kissing balloon inflation	67.9% (95)	96.8% (152)	< 0.0001	55.3% (168)	95.7% (313)	< 0.0001
Post-dilation	67.9% (95)	86.6% (136)	0.0001	68.4% (208)	84.4% (276)	< 0.0001
Balloon diameter, mm	3.76 ± 0.41	3.92 ± 0.56	0.05	3.88 ± 0.49	3.89 ± 0.54	0.65
Maximum inflation pressure, atm	17.8 ± 4.3	17.6 ± 4.2	0.57	17.5 ± 4.0	16.9 ± 4.5	0.06
Procedural complications*	2.9% (4)	2.6% (4)	1.00	1.0% (3)	3.4% (11)	0.04
IABP utilization	10.7% (15)	17.8% (28)	0.08	7.2% (22)	14.7% (48)	0.003
Procedure success	98.6% (138)	100.0% (157)	0.22	97.0% (295)	99.7% (326)	0.009

Values are mean ± SD or % (n). *Procedural complications including thrombosis, dissection, slow/no flow, severe spasm, and perforation.

*IABP* intra-aortic balloon pump, *IVUS* intravascular ultrasound; other abbreviations as in Table 1.

### Comparison of clinical outcomes between simple and complex LM bifurcation groups

**Figure 2:**
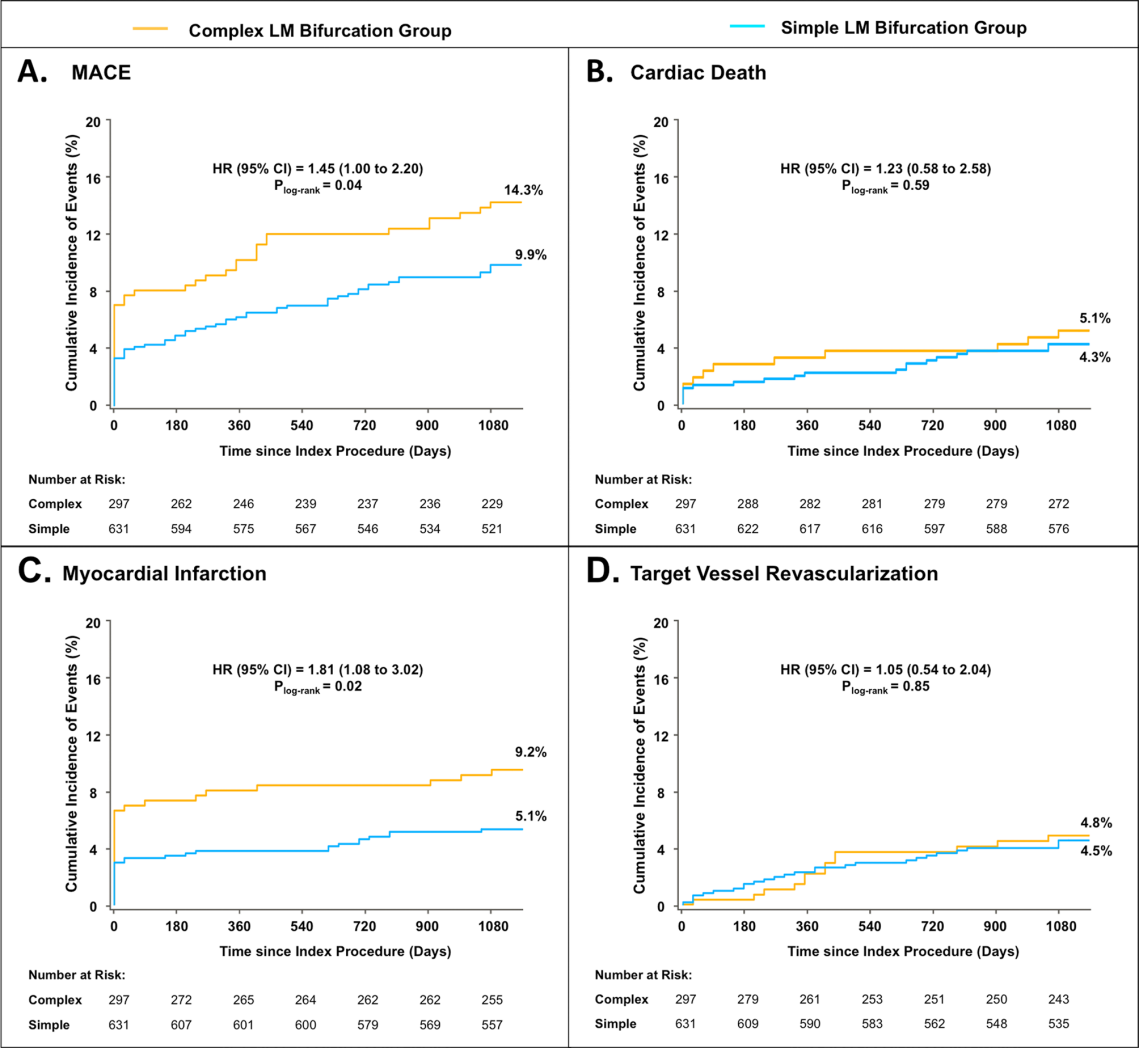
Time-to-event curves for 3-year clinical outcomes between complex and simple LM bifurcation lesion group. Hazard ratios are complex LM bifurcation group compared with simple LM bifurcation group. Major adverse cardiac event was defined as a composite of cardiac death, myocardial infarction, or target vessel revascularization. *MACE* major adverse cardiac events, *LM* left main, *HR* hazard ratio, *CI* confidence interval.

Patients in complex LM bifurcation group had significantly higher 30-day MACE rate compared with simple bifurcation group (7.8% vs. 4.0%, p = 0.01), which was mainly driven by increased MI rate (7.1% vs. 3.3%, p = 0.01). The difference in MACE rate between groups continued to 1 year (10.3% vs. 6.4%, p = 0.04) and numerically to 3 years (14.2% vs. 10.1%, p = 0.07), which was also mainly driven by significantly higher risk of MI (7.8% vs. 3.7%, p = 0.006 at 1 year and 9.4% vs. 5.3%, p = 0.02 at 3 years). Rates of cardiac death, TVR, as well as stent thrombosis were statistically comparable between groups (Table 4; Fig. 2). According to a Cox survial regression analysis, complex LM bifurcation lesion and lesion failure were independent risk factors for MACE (SI Table S4).

**Table 4:** Clinical Outcomes in simple and complex groups.

	Complex LM bifurcation group	Simple LM bifurcation group	p
**At 30 days**	**N = 297**	**N = 631**	
All-cause death	1.7% (5)	1.0% (6)	0.34
Cardiac death	1.4% (4)	1.0% (6)	0.73
MI	7.1% (21)	3.3% (21)	0.01
Periprocedural MI	6.7% (20)	2.7% (17)	0.003
Target-vessel related	7.1% (21)	3.3% (21)	0.01
Any revascularization	1.0% (3)	1.1% (7)	1.00
TVR	0.3% (1)	0.6% (4)	1.00
TLR	0.3% (1)	0.5% (3)	1.00
Definite/probable ST	0.7% (2)	0.6% (4)	1.00
MACE	7.8% (23)	4.0% (25)	0.01
**At 1 year**	**N = 292**	**N = 629**	
All-cause death	3.4% (10)	1.9% (12)	0.16
Cardiac death	2.4% (7)	1.6% (10)	0.40
MI	7.8% (23)	3.7% (23)	0.006
Target-vessel related	7.5% (22)	3.7% (23)	0.01
Any revascularization	7.5% (22)	4.5% (28)	0.05
TVR	2.1% (6)	2.4% (15)	0.75
TLR	2.7% (8)	2.4% (15)	0.75
Definite/probable ST	1.4% (4)	1.3% (8)	1.00
MACE	10.3% (30)	6.4% (40)	0.04
**At 3 years**	**N = 288**	**N = 605**	
All-cause death	5.2% (15)	4.5% (27)	0.62
Cardiac death	3.8% (11)	3.1% (19)	0.60
MI	9.4% (27)	5.3% (32)	0.02
Target-vessel related	9.0% (26)	5.3% (32)	0.03
Any revascularization	11.1% (32)	7.8% (47)	0.10
TVR	4.5% (13)	4.5% (27)	0.97
TLR	4.2% (12)	3.0% (18)	0.36
Definite/probable ST	2.8% (8)	2.3% (14)	0.68
Acute/subacute	0.7% (2)	0.6% (4)	1.00
Late	0.7% (2)	0.6% (4)	1.00
Very late	1.4% (4)	1.0% (6)	0.60
MACE	14.2% (41)	10.1% (61)	0.07

Values are % (n). Major adverse cardiac events was defined as a composite of cardiac death, myocardial infarction (MI), or target vessel revascularization.

*TVR* target vessel revascularization, *TLR* target lesion revascularization, *ST* stent thrombosis, *MACE* major adverse cardiac events; other abbreviations as in Table 1.

### Comparison of clinical outcomes between 1- or 2-stent groups

**Figure 3:**
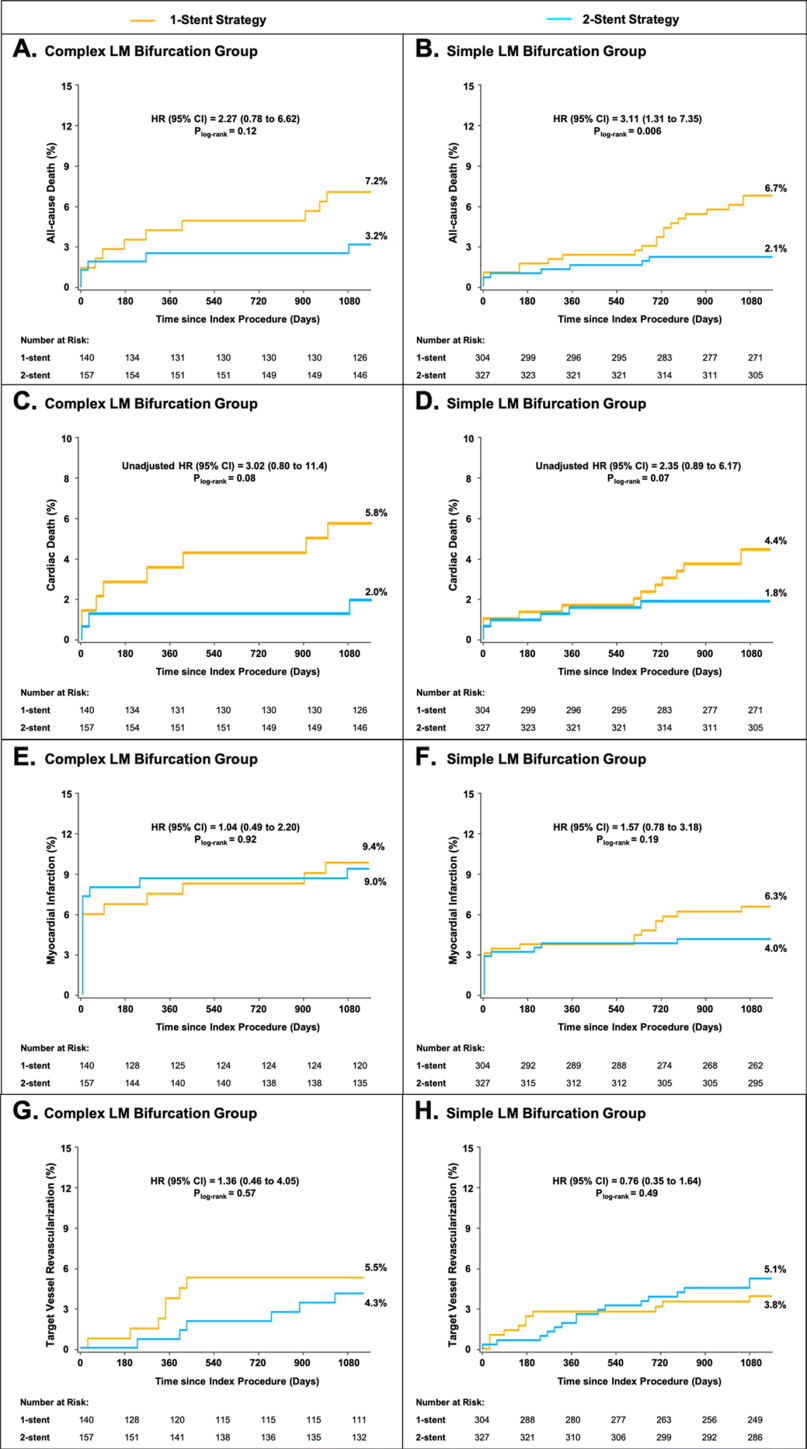
Survival curves for patients receiving 1- or 2-stent strategy through 3 years. Abbreviations as in Figs. 1 and 2.

As shown in Table 5, patients in the 2-stent strategy group had numerically lower rate of 3-year cardiac death events in both complex LM bifurcation group (2.0% vs. 5.9%, p = 0.08) and simple LM bifurcation group (1.9% vs. 4.5%, p = 0.07). Time-to-event curves showed that in complex LM bifurcation group, cardiac death event was higher with 1-stent strategy within 3 months and annually increased to 3 years; inversely, in simple LM bifurcation group the curves were similar at the beginning 2 years and principally separated between 2 and 3 years (Fig. 3), which was mainly due to higher incidence of very late ST event with 1-stent strategy (2.1% vs. 0%, p = 0.01). After IPTW adjustment, the incidence of cardiac death was significantly lower with 2-stent strategy in both complex and simple LM bifurcation group (hazard ratio [HR] 2.29, 95% confidence interval [CI] 1.41–3.75, p = 0.009 in complex group; HR 5.08, 95% CI 1.35–19.2, p = 0.02 in simple group). Other clinical events including all-cause death, MI as well as TVR was similar between 1- or 2-stent strategy before or after IPTW adjustment (Fig. 3, SI Figure S1). Whilst, age, left ventricular ejection fraction < 40%, and 1-stent strategy were independent risk factors for 3-year cardiac death (SI Table S4).

**Table 5:** Clinical Outcomes of 1-Stent and 2-Stent Strategy by LM Bifurcation Group.

	Complex LM bifurcation group			Simple LM bifurcation group		
	1-stent strategy	2-stent strategy	p	1-stent strategy	2-stent strategy	p
**At 30 days**	**N = 140**	**N = 157**		**N = 304**	**N = 327**	
All-cause death	1.4% (2)	1.9% (3)	1.00	1.0% (3)	0.9% (3)	1.00
Cardiac death	1.4% (2)	1.3% (2)	1.00	1.0% (3)	0.9% (3)	1.00
MI	6.5% (9)	7.6% (12)	0.70	3.3% (10)	3.4% (11)	0.96
Periprocedural MI	7.0% (11)	6.4% (9)	0.84	2.8% (9)	2.6% (8)	0.93
Target-vessel related	6.5% (9)	7.6% (12)	0.70	3.3% (10)	3.4% (11)	0.96
Any revascularization	0.7% (1)	1.3% (2)	1.00	2.0% (6)	0.3% (1)	0.06
TVR	0.7% (1)	0% (0)	0.47	1.0% (3)	0.3% (1)	0.36
TLR	0.7% (1)	0% (0)	0.47	1.0% (3)	0% (0)	0.11
Definite/probable ST	0% (0)	1.3% (2)	0.50	0.7% (2)	0.6% (2)	1.00
MACE	7.9% (11)	7.6% (12)	0.93	4.0% (12)	4.0% (13)	0.99
**At 1 year**	**N = 137**	**N = 155**		**N = 303**	**N = 326**	
All-cause death	4.4% (6)	2.6% (4)	0.52	2.3% (7)	1.5% (5)	0.48
Cardiac death	3.7% (5)	1.3% (2)	0.26	1.7% (5)	1.5% (5)	1.00
MI	7.3% (10)	8.4% (13)	0.73	3.6% (11)	3.7% (12)	0.97
Target-vessel related	7.3% (10)	7.7% (12)	0.89	3.6% (11)	3.7% (12)	0.97
Any revascularization	8.0% (11)	7.1% (11)	0.76	5.3% (16)	3.7% (12)	0.33
TVR	3.7% (5)	0.7% (1)	0.10	2.6% (8)	2.2% (7)	0.69
TLR	2.9% (4)	2.6% (4)	1.00	2.6% (8)	2.2% (7)	0.69
Definite/probable ST	1.5% (2)	1.3% (2)	1.00	1.3% (4)	1.2% (4)	1.00
MACE	12.4% (17)	8.4% (13)	0.26	6.2% (19)	6.4% (21)	0.93
**At 3 years**	**N = 136**	**N = 152**		**N = 292**	**N = 313**	
All-cause death	7.4% (10)	3.3% (5)	0.12	6.9% (20)	2.2% (7)	0.006
Cardiac death	5.9% (8)	2.0% (3)	0.08	4.5% (13)	1.9% (6)	0.07
MI	9.6% (13)	9.2% (14)	0.92	6.5% (19)	4.2% (13)	0.20
Target-vessel related	9.6% (13)	8.6% (13)	0.77	6.5% (19)	4.2% (13)	0.20
Any revascularization	11.0% (15)	11.2% (17)	0.97	8.2% (24)	7.4% (23)	0.69
TVR	5.2% (7)	4.0% (6)	0.62	3.8% (11)	5.1% (16)	0.42
TLR	3.7% (5)	4.6% (7)	0.69	2.7% (8)	3.2% (10)	0.74
Definite/probable ST	3.7% (5)	2.0% (3)	0.48	3.4% (10)	1.3% (4)	0.08
Acute/subacute	0% (0)	1.3% (2)	0.50	0.7% (2)	0.6% (2)	1.00
Late	1.4% (2)	0% (0)	0.22	0.7% (2)	0.6% (2)	1.00
Very late	2.2% (3)	0.7% (1)	0.35	2.1% (6)	0% (0)	0.01
MACE	16.2% (22)	12.5% (19)	0.37	10.6% (31)	9.6% (30)	0.67

Values are % (n). Abbreviations as in Tables 1 and 3.

## Discussion

The present study, a dedicated LM-PCI registry with largest number of true LM bifurcation lesions, systematic detailed LM intervention information collected to evaluate single or 2-stent treatment strategies, and long-term follow-up, demonstrated: (1) PCI in patients with complex LM bifurcation lesions as identified by DEFINITION criteria was associated with significantly higher risk of MACE, which was mainly driven by increased MI; (2) different to the non-LM bifurcations, 2-stent strategy may be beneficial on cardiac mortality for treatment of patients with true LM bifurcations.

### PCI treatment for true LM bifurcation lesions

Long-term results of the SYNTAX (Synergy Between Percutaneous Coronary Intervention With Taxus and Cardiac Surgery)^14^ and PRECOMBAT (Premier of Randomized Comparison of Bypass Surgery vs. Angioplasty Using Sirolimus-Eluting Stent in Patients with Left Main Coronary Artery Disease)^15^ randomized trials paved the way for PCI of LM disease in European guidelines, and recent EXCEL (Everolimus-Eluting Stents or Bypass Surgery for Left Main Coronary Artery Disease) trial^16^ underscored benefits of interventional treatment of selected LM patients. It comes to an agreement that in LM bifurcation patients with high surgical risk or with low or intermediate coronary anatomy complexity, PCI is a preferred treatment of choice^17^. In the present study, 3-year MACE rate was 11.4% in patients with true LM bifurcation patients following PCI treatment, which is acceptable and comparable with previous reports^9,18^, thereby providing further evidence that PCI is a favorable alternative for LM disease in selected patients even for true LM distal bifurcations with acceptable long-term outcomes.

### Complexity differentiation of true LM bifurcation lesions

Differentiation of bifurcation lesion complexity is aimed at informing precise and personalized treatment ultimately reducing SB occlusion and short- and long-term adverse clinical events. The Medina classification^10^ is although easy to remember and recommended for routine use, it has not usually been used for risk prediction. In the present study, DEFINITION criteria appeared useful in identifying complex LM bifurcation lesions. Patients identified with complex LM bifurcation lesions had significantly higher MACE rate up to 1 year and numerically at 3 years, mainly driven by significantly higher MI risk up to 3 years. Although acute procedural success rate was statistically similar between complex and simple LM bifurcation group, SB patency might be adversely affected during procedure as suggested by the difference of periprocedural MI rates. It is not surprising that true LM bifurcation lesion with complex anatomy requires more challenging procedure and likely linked to final TIMI decrease or side branch occlusion. It had been proven that both impairment of flow in coronary side branches and distal embolization of atheromatous material contribute to myocardial necrosis during PCI^19^. In this retrospective study, treatment choice of 1 or 2-stent strategy was similar irrespective of the LM lesion complexity, which was a daily clinical practice reflect when there was no tool for complexity classification and risk prediction. That in a sense proved the necessity of a scoring system for lesion evaluation.

### 1- versus 2-stent strategy for true LM bifurcation lesions

For treatment of distal LM bifurcation lesions, there is a debate on whether 1- or 2-stent strategy would better benefit long-term clinical outcomes. Cho et al. presented findings on 1,353 LM bifurcation patients highlighting more favorable 3-year MACE rate for 1-stent strategy especially with early-generation stents. Although the study enrolled a large cohort of patients, only 35.8% (484) had true LM bifurcation lesions, and 2-stent strategy was performed more frequently in patients with true bifurcation lesions and a more severe and longer lesion on SB^7,20^. In COBIS (Coronary Bifurcation Stenting) Registry II, 3-year clinical outcomes were worse after treatment of patients with distal LM bifurcation lesions with a 2-stent strategy, which was unanimously selected for patients with more complex bifurcation lesion anatomy and diffuse atherosclerotic involvement of both MV and SB^8^. In DKCRUSH-V (Double Kissing and Double Crush Versus Provisional T Stenting Technique for the Treatment of Unprotected Distal Left Main True Bifurcation Lesions: A Randomized, International, Multi-Center Clinical Trial) study, 482 patients with true LM bifurcation were randomized to DK crush or provisional stenting strategy. DK crush technique provided better prognosis including significantly lower composite endpoint of target lesion failure and stent thrombosis. On other hand, one-half of the patients in the provisional group received a second stent mainly due to complications during procedure^9,21^. Therefore, it appears important to select individualized treatment based on lesion anatomy; implantation of a second stent following a failed 1-stent strategy might be associated with worse prognosis. Consistently expert opinion also recommends choosing treatment strategy before the procedure, with optimal preparation before stenting coupled with kissing balloon inflations followed by final proximal optimization technique^5^. Another ongoing two pivotal randomized trials (EBC MAIN [European Bifurcation Club Left Main Study], NCT02497014 and DEFINITION II, NCT02284750) will provide more evidence^22,23^.

In the present study, 2-stent strategy yielded lower rate of 3-year cardiac death among patients with true LM bifurcation lesions regardless of lesion complexity per DEFINITION criteria. However, there were still differences between complex and simple bifurcation population. In the complex group, 2-stent strategy showed its benefit since the very early time and maintaining to 3 years, which can be easily explained that true LM bifurcation lesions with complex anatomy requires adequate treatment to avoid jailed side branch or severe residual stenosis. In contrast, in simple LM bifurcation group, even though procedural success was significantly lower with 1-stent strategy, early events were similar between the 2 strategies. Cardiac death principally differentiated after 2 years, which was mainly due to very late stent thrombosis. The final Cox regression analysis was inconsistent with widespread agreements that major risk factors for long-term cardiac death were age and lower left ventricular ejection fraction (< 40%). Besides, for true LM bifurcation patients, 2-stent strategy would befinite more.

For true LM bifurcation lesions, both 1- and 2-stent strategy showed no improvement in terms of periprocedural MI. That was on the one hand, more complications occurred with 2-stent strategy in the simple LM bifurcation group, mostly due to failed provisional strategy and thus a bail-out stent in the SB was needed. On the other hand, for 2-stent approach, precise evaluate bifurcation angle and sidebranch burden then chose an appropriate approach (culotte, mini-crush, or DK crush) was necessary to reduce complications. More patients underwent final-kissing balloon and IVUS guidance might be another reason for a better prognosis with 2-stent strategy. Results of the study support the concept that a well-planned 2-stent strategy (in case of complications caused by provisional 1-stent approach) might reduce periprocedural risk for true LM bifurcation lesions, and appropriate approach (e.g. DK crush) performed by high-volume operators would benefit more^9,21^. Finally, although the present results were inconsistent with previous findings from morjarity of other bifurcation (with or without left main) studies, conclusion of this restrospective cohort only focuses on true left main bifurcation lesions (Medina 1,1,1 or 0,1,1). It’s not suitable to generalize this to overall bifurcation patients, which we still believe a simpler approach—provisional stenting—should be used.

In the present study, crush technique was used in most 2-stent strategy cases, while T-stent strategy was used for patients with higher bifurcation angles. Rate of procedural complications was relatively low, mainly because of using 2-stent strategy was decided prospectively in contrast to bailout stenting followed failed 1-stent strategy. An advanced 2-stent strategy (e.g., DK crush technique) in the hands of proficient high-volume operators^24^ appears to provide more benefits for patients with true LM bifurcation lesions.

### Limitations

The study has the limitations inherent to its retrospective design, which might have introduced selection bias; however, we used an IPTW method to minimize such possibility. Secondly, the single-center scope of the study might limit external validity of the major findings. Thirdly, patient’s enrollment of the LM registry study begins since 2004, PCI strategy, IVUS usage or stent generation has been evolved greatly. However, during the early times, LM cases in this center were mainly performed by experienced operators, which could also reflect a high-quality PCI result. Fourthly, the angiogram characteristics of the MV or SB were evaluated visually rather than via quantitative coronary angiography by an independent core lab, which might attenuate precision. However, operators routinely rely on visual estimation for treatment selection, which makes our findings more valuable for daily practice. Finally, rates of events especially those for cardiac death were relatively low, which might lead to low statistical power.

### Conclusions

In the present study on a large cohort of consecutive patients with true LM bifurcation lesion, use of the complex bifurcation lesion criteria established in DEFINITION study appears to allow risk stratification and long-term MACE prediction. Two-stent technique yielded numerically lower 3-year cardiac death rate among patients with true LM bifurcations regardless of lesion complexity.

## Supplementary information

Supplementary file

## Abbreviations

DES: Drug-eluting stents
DK: Double-kissing
LM: Left main
MACE: Major adverse cardiac events
MI: Myocardial infarction
MV: Main vessel
PCI: Percutaneous coronary intervention
PS: Propensity score
SB: Side branches
TVR: Target vessel revascularization

## References

[1] Sawaya FJ (2016). Contemporary approach to coronary bifurcation lesion treatment. JACC Cardiovasc. Interv..

[2] Leesar MA, Hakeem A, Azarnoush K, Thuesen L (2015). Coronary bifurcation lesions: Present status and future perspectives. Int. J. Cardiol..

[3] Ragosta M (2006). Prevalence of unfavorable angiographic characteristics for percutaneous intervention in patients with unprotected left main coronary artery disease. Catheter Cardiovasc. Interv..

[4] Bittl JA (2015). Treatment of bifurcation lesions: Less is more. J. Am. Coll. Cardiol..

[5] Banning AP (2019). Percutaneous coronary intervention for obstructive bifurcation lesions: The 14th consensus document from the European Bifurcation Club. EuroIntervention.

[6] Ford TJ (2018). Single- versus 2-stent strategies for coronary bifurcation lesions: A systematic review and meta-analysis of randomized trials with long-term follow-up. J. Am. Heart Assoc..

[7] Cho S (2018). Long-term clinical outcomes and optimal stent strategy in left main coronary bifurcation stenting. JACC Cardiolvasc. Interv..

[8] Song YB (2014). Differential prognostic impact of treatment strategy among patients with left main versus non-left main bifurcation lesions undergoing percutaneous coronary intervention: Results from the COBIS (Coronary Bifurcation Stenting) Registry II. JACC Cardiovasc. Interv..

[9] Chen SL (2017). Double kissing crush versus provisional stenting for left main distal bifurcation lesions: DKCRUSH-V randomized trial. J. Am. Coll. Cardiol..

[10] Chen SL (2014). Impact of the complexity of bifurcation lesions treated with drug-eluting stents: The DEFINITION study (Definitions and impact of complEx biFurcation lesIons on clinical outcomes after percutaNeous coronary IntervenTIOn using drug-eluting steNts). JACC Cardiovasc. Interv..

[11] Medina A, Suárez de Lezo J, Pan M (2006). A new classification of coronary bifurcation lesions [Article in Spanish]. Rev. Esp. Cardiol..

[12] Lansky A (2009). Quantitative angiographic methods for bifurcation lesions: a consensus statement from the European Bifurcation Group. Cathether Cardiovasc. Interv..

[13] Cutlip DE (2007). Clinical end points in coronary stent trials: A case for standardized definitions. Circulation.

[14] Mohr FW (2013). Coronary artery bypass graft surgery versus percutaneous coronary intervention in patients with three-vessel disease and left main coronary disease: 5-year follow-up of the randomised, clinical SYTNAX trial. Lancet.

[15] Ahn JM (2015). Randomized trial of stents versus bypass surgery for left main coronary artery disease: 5-year outcomes of the PRECOMBAT study. J. Am. Coll. Cardiol..

[16] Stone GW (2016). Everolimus-eluting stents or bypass surgery for left main coronary artery disease. N. Engl. J. Med..

[17] Tam DY (2019). Modality selection for the revascularization of left main disease. Can. J. Cardiol..

[18] Pavani M (2018). Long-term outcomes of different two-stent technique with second-generation drug-eluting stents for unprotected left main bifurcation disease: Insights from the FAILS-2 study. J. Invas. Cardiol..

[19] Porto I (2006). Plaque volume and occurrence and location of periprocedural myocardial necrosis after percutaneous coronary intervention: Insights from delayed-enhancement magnetic resonance imaging, thrombolysis in myocardial infarction myocardial perfusion grade analysis, and intravascular ultrasound. Circulation.

[20] Chevalier B (2018). Left main bifurcation angioplasty: Are 2 stents one too many?. JACC Cardiovasc. Interv..

[21] Brilakis ES, Burke MN, Banerjee S (2017). DK-Crush should become preferred strategy for treating unprotected LM bifurcation lesions: No pain, no gain. J. Am. Coll. Cardiol..

[22] Chieffo A, Hildick-Smith D (2016). The European Bifurcation Club Left Main Study (EBC MAIN): Rationale and design of an international, multicenter, randomised comparison of two stent strategies for the treatment of left main coronary bifurcation disease. EuroIntervention.

[23] Zhang JJ (2018). Treatment effects of systematic two-stent and provisional stenting techniques in patients with complex coronary bifurcation lesions: Rationale and design of a prospective, randomised and multicentre DEFINITION II trial. BMJ Open..

[24] Xu B (2016). Impact of operator experience and volume on outcomes after left main coronary artery percutaneous coronary intervention. JACC Cardiovasc. Interv..

