# Supplementary material for: Validation of bifurcation DEFINITION criteria and comparison of stenting strategies in true left main bifurcation lesions
Source: Sci Rep. 2020 Jun 26;10:10461. doi: 10.1038/s41598-020-67369-9 (PMC7320001; doi:10.1038/s41598-020-67369-9)

**Supplementary Appendix**

**Validation of Bifurcation DEFINITION Criteria and Comparison of Stenting Strategies in True Left Main Bifurcation Lesions**

Juan Wang, MD,^1^ Changdong Guan, MSc,^2^ Jue Chen, MD,^1^ Kefei Dou, MD,^1,3^ Yida Tang, MD,^1,3^ Weixian Yang, MD,^1,3^ Yanpu Shi, MD,^1^ Fenghuan Hu, MD,^1^ Lei Song, MD,^1^ Jiansong Yuan, MD,^1^ Jingang Cui, MD,^1^ Min Zhang, BS,^4^ Shuang Hou, MMed,^4^ Yongjian Wu, MD,^1,3^ Yuejin Yang, MD,^1,3^ Shubin Qiao, MD,^1,3^ Bo Xu, MBBS,^2,3^

^1^Department of Cardiology, Fu Wai Hospital, National Center for Cardiovascular Diseases, Chinese Academy of Medical Sciences, Beijing, China; ^2^Catheterization Laboratories, Fu Wai Hospital, National Center for Cardiovascular Diseases, Chinese Academy of Medical Sciences, Beijing, China; ^3^National Clinical Research Center for Cardiovascular Diseases, Beijing, China; ^4^CCRF (Beijing) Inc, Beijing, China.

**Brief title:** Complexity & Strategy in True LM Bifurcation

**Table S1. Standardized Differences With or Without PS Match in Complex and Non-Complex LM Bifurcation Populations Receiving 1- or 2-Stent Strategy**

| **Complex LM Bifurcation Group** | | | **Simple LM Bifurcation Group** | | |
| --- | --- | --- | --- | --- | --- |
| **Variables** | **Standardized Difference Without Adjustment** | **Standardized Difference With PS Match Adjustment** | **Variables** | **Standardized Difference Without Adjustment** | **Standardized Difference With PS Match Adjustment** |
| Main vessel lesion length | 0.22 | 0.05 | Gender | 0.08 | 0.02 |
| Main vessel reference vessel diameter | 0.22 | 0.04 | Main vessel lesion length | 0.16 | 0.03 |
| Side branch lesion length | 0.25 | 0.07 | Main vessel diameter stenosis | 0.25 | 0.02 |
| Side branch reference vessel diameter | 0.52 | 0.04 | Side branch lesion length | 0.18 | 0.04 |
| Side branch diameter stenosis | 0.12 | 0.01 | Side branch reference vessel diameter | 0.30 | 0.09 |
| Medina type | 0.25 | 0.04 | Side branch diameter stenosis | 0.62 | 0.005 |
| Transradial Approach | 0.06 | 0.02 | Medina type | 0.43 | 0.04 |
| Guidance with IVUS | 0.43 | 0.03 | Transradial Approach | 0.32 | 0.02 |
| IABP utilization | 0.20 | 0.01 | IABP utilization | 0.24 | 0.01 |
| Procedural success | 0.17 | 0.12 | Procedural success | 0.21 | 0.12 |

PS = propensity score; LM = left main. SYNTAX = synergy between percutaneous coronary intervention with TAXUS and cardiac surgery.

**Table S2. Baseline Patient and Lesion Characteristics**

|  | **Complex LM Bifurcation Group**  **N=297** | **Simple LM Bifurcation Group**  **N=631** | **p** |
| --- | --- | --- | --- |
| Age, years | 62.0 ± 9.9 | 60.3 ± 10.9 | 0.02 |
| Male | 80.1% (238) | 80.2% (506) | 0.98 |
| Body mass index, kg/m^2^ | 25.8 ± 3.1 | 25.8 ± 3.2 | 0.93 |
| Diabetes mellitus | 29.3% (87) | 28.4% (179) | 0.77 |
| Insulin-requiring | 13.5% (10) | 13.2% (22) | 0.93 |
| Hypertension | 59.6% (177) | 56.9% (359) | 0.44 |
| Hyperlipidemia | 58.9% (175) | 52.8% (333) | 0.08 |
| Family history of coronary artery disease | 18.5% (55) | 17.1% (108) | 0.60 |
| Current tobacco use | 32.0% (95) | 34.6% (218) | 0.44 |
| Previous myocardial infarction | 33.7% (100) | 30.1% (190) | 0.28 |
| Previous stroke | 10.4% (31) | 8.1% (51) | 0.24 |
| Peripheral arterial disease | 7.4% (22) | 3.5% (22) | 0.009 |
| Previous percutaneous coronary intervention | 31.0% (92) | 28.5% (180) | 0.44 |
| Creatinine clearance rate | 89.6 ± 26.9 | 90.7 ± 26.7 | 0.45 |
| Unstable angina | 58.6% (174) | 65.9% (416) | 0.03 |
| Left ventricular ejection fraction, % | 61.5 ± 8.5 | 62.9 ± 7.5 | 0.11 |
| Coronary artery disease extent |  |  | <0.0001 |
| Isolated LM | 0% (0) | 1.0% (6) |  |
| LM+1VD | 0.7% (2) | 3.3% (21) |  |
| LM+2VD | 37.7% (112) | 49.3% (311) |  |
| LM+3VD | 61.6% (183) | 46.4% (293) |  |
| SYNTAX Score | 28.4 ± 6.1 | 25.7 ± 6.9 | <0.0001 |
| Classification |  |  | <0.0001 |
| Medina 1,1,1 | 88.6% (263) | 76.1% (480) |  |
| Medina 0,1,1 | 11.5% (34) | 23.9% (151) |  |
| Chronic total occlusion | 5.1% (15) | 2.5% (16) | 0.05 |
| Moderate to severe calcification | 25.6% (76) | 6.8% (43) | <0.0001 |
| Thrombus-containing | 4.4% (13) | 0.6% (4) | 0.0004 |
| Multiple lesions^*^ | 100% (297) | 99.5% (628) | 0.56 |
| Main vessel |  |  |  |
| Lesion length, mm | 39.5 ± 19.7 | 21.1 ± 15.0 | <0.0001 |
| Lesion length ≥25 mm | 84.5% (251) | 21.9% (138) | <0.0001 |
| Reference vessel diameter, mm | 3.48 ± 0.50 | 3.69 ± 0.50 | <0.0001 |
| Reference vessel diameter <3.0 mm | 6.7% (20) | 3.2% (20) | 0.01 |
| Reference vessel diameter <2.5 mm | 0.7% (2) | 0% (0) | 0.10 |
| Diameter stenosis, % | 86.3 ± 8.4 | 83.1 ± 10.6 | <0.0001 |
| Diameter stenosis ≥70% | 98.3% (292) | 93.7% (591) | 0.002 |
| Side branch |  |  |  |
| Lesion length | 30.9 ± 21.8 | 20.4 ± 16.1 | <0.0001 |
| Lesion length ≥10 mm | 100.0% (297) | 82.7% (521) | <0.0001 |
| Reference vessel diameter | 2.91 ± 0.41 | 3.01 ± 0.42 | <0.0001 |
| Reference vessel diameter ≥2.5 mm | 97.0% (288) | 97.5% (614) | 0.67 |
| Diameter stenosis, % | 85.1 ± 9.0 | 74.1 ± 15.4 | <0.0001 |
| Diameter stenosis ≥70% | 100.0% (297) | 70.8% (447) | <0.0001 |
| Bifurcation angle <45^°^ | 2.5% (6) | 0.9% (4) | 0.18 |

Values are mean ± SD or % (n). ^*^Multiple lesions included multiple-vessel disease (defined as ≥70% stenosis in at least 1 major epicardial vessel and ≥50% stenosis in at least 1 other major vessel) or ≥2 lesions separated by at least a 5-mm normal segment in the target vessel. LM = left main; VD = vessel disease; SYNTAX = synergy between percutaneous coronary intervention with TAXUS and cardiac surgery.

**Table S3. Procedural Characteristics and Results**

|  | **Complex LM Bifurcation Group**  **N=297** | **Simple LM Bifurcation Group**  **N=631** | **p** |
| --- | --- | --- | --- |
| Transradial approach | 62.3% (185) | 61.5% (388) | 0.82 |
| Guidance with IVUS | 41.8% (124) | 42.0% (265) | 0.94 |
| Stent implantation |  |  |  |
| Number of stents per patient | 2.87 ± 1.17 | 2.17 ± 1.04 | <0.0001 |
| Stent diameter, mm | 3.27 ± 0.45 | 3.43 ± 0.49 | <0.0001 |
| Stent length, mm | 47.8 ± 23.8 | 29.6 ± 18.0 | <0.0001 |
| Maximum inflation pressure, atm | 15.9 ± 3.0 | 16.0 ± 3.0 | 0.61 |
| 2-Stent Strategy | 52.9% (157) | 51.8% (327) | 0.77 |
| Crush | 40.4% (120) | 34.5% (218) | 0.08 |
| Mini crush | 31.6% (94) | 28.4% (179) | 0.31 |
| DK crush | 8.8% (26) | 6.2% (39) | 0.15 |
| T-Stent | 5.1% (15) | 6.7% (42) | 0.34 |
| V- or Kissing Stent | 2.4% (7) | 6.2% (39) | 0.01 |
| Culotte | 5.1% (15) | 4.4% (28) | 0.68 |
| Final kissing balloon inflation | 83.2% (247) | 76.2% (481) | 0.02 |
| Post-dilation | 77.8% (231) | 76.7% (484) | 0.72 |
| Balloon diameter, mm | 3.86 ± 0.51 | 3.59 ± 0.52 | 0.28 |
| Maximum inflation pressure, atm | 17.7 ± 4.2 | 17.2 ± 4.3 | 0.33 |
| Procedural complications^*^ | 2.7% (8) | 2.2% (14) | 0.66 |
| IABP utilization | 14.5% (43) | 11.1% (70) | 0.14 |
| Procedure success | 99.3% (295) | 98.4% (621) | 0.36 |

Values are mean ± SD or % (n). ^*^Procedural complications including thrombosis, dissection, slow/no flow, severe spasm, and perforation. IABP = intra-aortic balloon pump; IVUS = intravascular ultrasound.

**Table S4. Cox Survival Regression Analysis for 3-Year MACE and Cardiac Death**

|  | **HR (95% CI)** | **p** |
| --- | --- | --- |
| **MACE** |  |  |
| Age (continues increase) | 1.01 (0.99 to 1.03) | 0.21 |
| Female | 1.14 (0.73 to 1.78) | 0.58 |
| Diabetes | 0.76 (0.49 to 1.18) | 0.23 |
| Left ventricular ejection fraction < 40% | 2.90 (0.91 to 9.22) | 0.07 |
| Residual SYNTAX score > 8 | 1.31 (0.85 to 2.01) | 0.22 |
| 2-stent strategy (vs. 1-sent strategy) | 1.00 (0.69 to 1.46) | 0.99 |
| Complex LM bifurcation lesion (as defined by DEFINITION criteria) | 1.53 (1.05 to 2.24) | 0.03 |
| Lesion failure | 3.09 (1.09 to 8.79) | 0.03 |
| **Cardiac death** |  |  |
| Age (continues increase) | 1.04 (1.01 to 1.08) | 0.02 |
| Female | 1.55 (0.68 to 3.57) | 0.30 |
| Diabetes | 0.45 (0.17 to 1.20) | 0.11 |
| Left ventricular ejection fraction < 40% | 14.9 (4.25 to 52.4) | <0.0001 |
| Residual SYNTAX score > 8 | 1.38 (0.63 to 3.02) | 0.43 |
| 2-stent strategy (vs. 1-sent strategy) | 0.38 (0.17 to 0.84) | 0.02 |
| Complex LM bifurcation lesion (as defined by DEFINITION criteria) | 1.17 (0.55 to 2.47) | 0.69 |
| Lesion failure | 1.51 (0.19 to 11.8) | 0.70 |

CI = confidence interval; HR = hazard ratio; other abbreviations as in Table S1.

**Figure S1. Adjusted Survival Curves for Patients Receiving 1- or 2-Stent Strategy Through 3 Years**

Hazard ratios are 1-stent strategy compared with 2-stent strategy. LM = left main; HR = hazard ratio; CI = confidence interval.


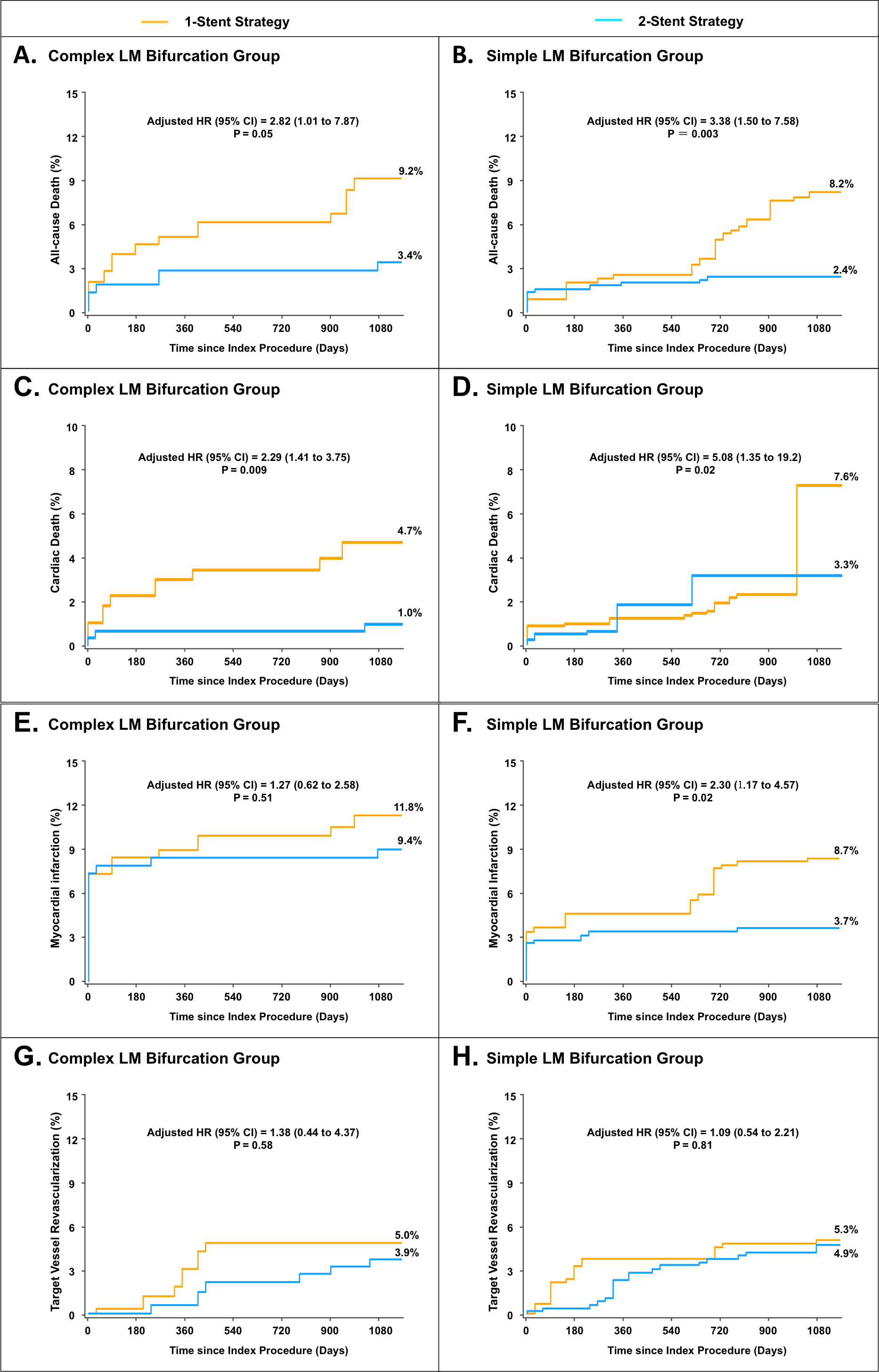

Supplement: Supplementary file 1 — Supplementary file [file 41598_2020_67369_MOESM1_ESM.docx]
